# Exploring the Influence of Gut Microbiome on Energy Metabolism in Humans

**DOI:** 10.1016/j.advnut.2023.03.015

**Published:** 2023-04-07

**Authors:** Julia Montenegro, Anissa M. Armet, Benjamin P. Willing, Edward C. Deehan, Priscila G. Fassini, João F. Mota, Jens Walter, Carla M. Prado

**Affiliations:** 1 Department of Agricultural, Food and Nutritional Science, University of Alberta, Edmonton, Alberta, Canada; 2 Department of Food Science and Technology, University of Nebraska, Lincoln, Nebraska, United States; 3 Nebraska Food for Health Center, University of Nebraska, Lincoln, Nebraska, United States; 4 Department of Internal Medicine, Division of Nutrology, Ribeirão Preto Medical School, University of São Paulo, Ribeirão Preto, São Paulo, Brazil; 5 School of Nutrition, Federal University of Goiás, Goiânia, Goiás, Brazil; 6 APC Microbiome Ireland, School of Microbiology, and Department of Medicine, University College Cork – National University of Ireland, Cork, Ireland

**Keywords:** Gut microbiome, energy metabolism, energy expenditure, energy harvest

## Abstract

The gut microbiome has a profound influence on host physiology, including energy metabolism, which is the process by which energy from nutrients is transformed into other forms of energy to be used by the body. However, mechanistic evidence for how the microbiome influences energy metabolism is derived from animal models. In this narrative review, we included human studies investigating the relationship between gut microbiome and energy metabolism —i.e., energy expenditure in humans and energy harvest by the gut microbiome. Studies have found no consistent gut microbiome patterns associated with energy metabolism, and most interventions were not effective in modulating the gut microbiome to influence energy metabolism. To date, cause-and-effect relationships and mechanistic evidence on the impact of the gut microbiome on energy expenditure have not been established in humans. Future longitudinal observational studies and randomized controlled trials utilizing robust methodologies and advanced statistical analysis are needed. Such knowledge would potentially inform the design of therapeutic avenues and specific dietary recommendations to improve energy metabolism through gut microbiome modulation.

**Statement of Significance**

The gut microbiome influence energy metabolism in the host, which has been shown in animal models. Here we review human studies that investigated the interaction between the gut microbiome and the host’s energy metabolism, and we developed a framework for future studies.

## Introduction

Energy metabolism is the process by which energy stored in macronutrients (i.e., carbohydrates, lipids, proteins, and alcohol) is transformed into other forms of energy (e.g., heat and adenosine triphosphate) [1, 2]. At the cellular level, energy metabolism refers to the pathways involved in substrate catabolism and oxidative phosphorylation [3], whereas at the whole-body level, it is the balance between an individual’s energy intake and energy expenditure [4]. Resting energy expenditure (REE), diet-induced thermogenesis, and the energy cost of physical activity are the main components of total energy expenditure (TEE) [5]. Diet independently influences energy metabolism because each nutrient has a different thermic effect (e.g., carbohydrates and protein require more energy to be metabolized than fat) [6].

**FIGURE 1:**
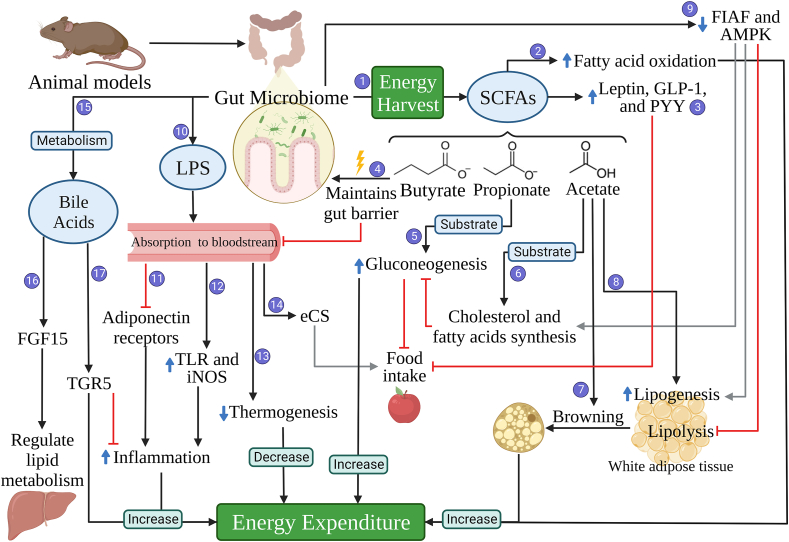
**Evidence for the importance of the gut microbiome in energy metabolism from animal models.** 1) The fermentation of dietary fiber produces short-chain fatty acids (SCFAs), increasing the availability of energy for the host; 2) SCFAs promote mitochondrial fatty acid oxidation and energy expenditure; 3) SCFAs modulate signaling pathways through activation of free fatty acid receptors, increasing secretion of leptin, glucagon-like peptide-1 (GLP-1), and peptide YY (PYY), leading to increased satiety and reduced energy intake; 4) Butyrate is an energy source for colonocytes and helps maintain gut barrier function by increasing the expression of tight junction proteins and mucus production, which reduces LPS absorption; 5) Propionate is used as a substrate for gluconeogenesis, which increases energy expenditure and reduces food intake and cholesterol synthesis; 6) Acetate is a substrate for cholesterol and fatty acid synthesis, which inhibits gluconeogenesis; 7) Acetate causes browning of white adipose tissue, which increases energy expenditure; 8) Acetate increases the expression of genes associated with lipogenesis; 9) The gut microbiome reduced the intestinal expression of fasting-induced adipose factor and reduces the release of adenosine monophosphate-activated protein kinase, which increases cholesterol and fatty acids synthesis, increases lipogenesis, and inhibits lipolysis; 10) The cell wall of gram-negative bacteria have lipopolysaccharides (LPS) which are proinflammatory endotoxins that can be absorbed; 11) Circulating LPS inhibits adiponectin receptors, which is anti-inflammatory; 12) Circulating LPS activates Toll-like receptors, which are associated with recognition of pathogens, and increases inducible nitric oxide synthase (iNOS), which is a biomarker of stress, both increase inflammation and leads to increased energy expenditure; 13) High LPS in the blood reduce thermogenesis and, thus, energy expenditure; 14) LPS activates the endocannabinoid system (cCS), increasing food intake; 15) The gut microbiome metabolizes bile acids to their unconjugated forms, altering their functions; 16) Different bile acids have different levels of activation of farnesoid X receptor, which upregulates the expression of fibroblast growth factor 15 (FGF15), which inhibits hepatic triglycerides and bile acid synthesis; 17) Different bile acids have different levels of activation of G-protein-coupled bile acid receptor (TGR5), which inhibits inflammatory pathways and increases energy expenditure. This evidence has been derived from animal models, which includes rodents (as shown in the figure) and other animals, such as pigs and zebrafish.

The gut microbiome (the microbial community that colonizes the intestinal tract) performs diverse functions that influence many aspects of host biology [7], including energy metabolism. The mechanisms from animal evidence are summarized in Figure 1. Those mechanisms involve energy harvest through the production of short-chain fatty acids (SCFAs) by fermentation of dietary fiber [[8], [9], [10], [11]] and the influence of gut microbes in inflammatory response [12, 13]. Although inflammation generally enhances energy expenditure [12], inflammation driven by lipopolysaccharides (LPS) and other microbial molecules, however, has been associated with greater adiposity and insulin resistance [14, 15]. Furthermore, bile acids derivatives produced by the gut microbiota function as signaling molecules, which may increase or decrease energy expenditure by regulating inflammation and hepatic lipid metabolism [15, 16].

The possibility of manipulating the microbiome to improve energy metabolism has generated great interest in the past 2 decades, especially in the context of obesity, and mechanisms by which the gut microbiome affects host energy metabolism have been primarily studied in this context [1, 14, 15, [17], [18], [19], [20], [21]]. However, most evidence gathered to date comes from animal models and in vitro characterizations, which may have limited translatability to humans. This narrative review aims to address this knowledge gap by reviewing the evidence from human trials exploring the relationship between the gut microbiome and energy metabolism. Our secondary goal was also to identify gaps and opportunities to facilitate the development of recommendations for modulating energy metabolism through gut microbiome.

## Methods

We searched for articles on MEDLINE and CINAHL databases between February and April 2021, with an updated search in September 2022. Search terms included “energy AND (metabolism OR balance OR expenditure OR yield OR harvest)” and “(gut OR gastrointestinal OR intestinal OR bowel OR colon) AND (microbiome OR microbiota OR microflora OR bacteria OR microbes).” We included only original studies in humans that analyzed gut microbiome and at least one of the variables related to energy metabolism; variables included: SCFAs, energy in stool, energy expenditure, and functional activity of the microbiome, which are further explained in the following section. Studies testing probiotics and antibiotics were not included. Articles written in English were included with no restrictions regarding time of publication, population, study design, or health conditions. Reported findings considered 2 possibilities: 1) nutrients affect energy metabolism directly because of diet-induced thermogenesis and may affect it indirectly by changing gut microbiome composition and its production of metabolites [22], and 2) such effects might alter host energy metabolism. BMI classifications (i.e., underweight, normal weight, overweight, and obesity) described in this review are based on the World Health Organization [23, 24] unless otherwise specified.

A total of 20 articles were included; Table 1 summarizes observational studies [[25], [26], [27], [28], [29], [30]], and Table 2 summarizes intervention studies with randomized controlled trials (parallel-arm [[31], [32], [33], [34], [35], [36], [37]] and crossover [[38], [39], [40], [41], [42]]) or single-arm interventions [43, 44].

**TABLE 1:** Summary of results from observational human studies assessing energy metabolism and the gut microbiome.

Reference	Study design	Assessments	Main outcomes
Bielik *et al.*, 2020 [25]	- Male athletes (*n* = 24) - Athletes with positive energy balance compared to athletes with isocaloric intake	- REE, TEE, TREE, and RER (metabolic cart) - Microbiome (metagenomic)	- No significant differences in REE and TEE
			- *Roseburia* correlated with body fat (r = 0.577, *P <* 0.01), energy intake (r = 0.446, *P <* 0.05), TREE (r = 0.500, *P <* 0.05), VO_2_rest (r = 0.432, *P <* 0.05), carbohydrate intake (r = 0.410, *P <* 0.05), and RER (r = -0.419, *P <* 0.05)
			- Alteromonadales correlated with VO_2_rest (r = -0.408, *P <* 0.05)
Boekhorst *et al.*, 2022 [26]	- Adults (*n* = 85) with overweight or obesity (BMI 25–35 kg/m^2^) - Gut microbiome enterotypes: *Bacteroides* (B-type), *Prevotella* (P-type), or *Rumminococcaceae* (R-type)	- Stool energy density and SCFAs - Microbiome (16S rRNA) - Intestinal transit time (radio-opaque marker) - Urine proteolytic metabolites	- B-type had lower stool energy density and shorter transit time than R-type (*P <* 0.05 and *P <* 0.001 respectively)
			- Transit time was correlated with stool energy density (r_s_ = 0.23, *P* = 0.027) and SCFAs (r not shown, *P <* 0.05)
			- R-type had higher stool branched SCFAs (isobutyrate, 2-methylbutyrate, and isovalerate) than B-type (*P <* 0.05)
			- P-type had higher levels of valereate and caproate than B-type (both *P <* 0.01)
			- R-type had higher levels of p-cresol sulfate (B-type *P <* 0.001, P-type *P <* 0.05), p-cresol glucuronide (B-type *P <* 0.01, P-type *P <* 0.05), and phenylacetylglutamine (B-type *P <* 0.001, P-type *P <* 0.01)
Ghosh *et al.*, 2014 [27]	- Children (≤60 mo old, *n* = 20) - Apparently healthy compared to borderline and severely malnourished	- Microbiome (metagenomic and functional capacity)	- Nutritional index was correlated with *Roseburia* (r_s_ = 0.51, *P* = 0.048), *Faecalibacterium* (r_s_ = 0.49, *P* = 0.003), *Butyrivibrio* (r_s_ = 0.52, *P* = 0.027), *Escherichia* (r_s_ = -0.59, *P* = 0.032), *Streptococcus* (r_s_ = -0.70, *P* = 0.019), *Shigella* (r_s_ = -0.51, *P* = 0.048), *Enterobacter* (r_s_ = -0.75, *P* = 0.032), and *Veillonella* (r_s_ = -0.80, *P* = 0.005)
			- Good nutritional status had over-representation of microbial categories of functional potential related to nutrient uptake and metabolism, and energy production and conversion.
			- Poor nutritional status had over-representation of microbial categories of functional potential related with virulence and bacterial pathogenesis
Goffredo *et al.*, 2016 [28]	- Children and adolescents (n = 84) - BMI classification comparison (non-obese, overweight, obese, and severely obese)	- Fasting plasma SCFAs - Carbohydrate oxidation by gut microbiome - Microbiome (16S rRNA)	- BMI was positively correlated with F/B ratio (*P* = 0.002) and Actinobacteria (*P* = 0.01); and negative correlation with Bacteroidetes (*P <* 0.001); r values not shown.
			- F/B ratio was correlated with total body fat (r = 0.187, *P* = 0.042), subcutaneous fat (r = 0.251, *P* = 0.032), and hepatic fat (r = 0.339, *P* = 0.002)
			- Bacteroidetes was correlated with total body fat (r = 0.209, *P* = 0.027), visceral fat (r = 0.250, *P* = 0.031), subcutaneous fat (r = 0.288, *P* = 0.012), and hepatic fat (r = 0.330, *P* = 0.003)
			- Actinobacteria was correlated with visceral fat (r = 0.250, *P* = 0.031).
			- Acetate was correlated with F/B ratio (r = 0.419, *P* = 0.001) and Bacteroidetes (r = 0.395, *P* = 0.001).
			- Total body fat, visceral fat, and subcutaneous fat was correlated with acetate (r = 0.283, *P* = 0.004, r = 0.329, *P* = 0.002, and r = 0.397, *P* = 0.001), propionate (r = 0.534, *P <* 0.001, r = 0.496, *P <* 0.001, and r = 0.571, *P <* 0.001), and butyrate (r = 0.555, *P <* 0.001, r = 0.455, *P <* 0.001, and r = 0.539, *P <* 0.001)
			- Hepatic lipogenesis was positively correlated with acetate (r = 0.67, *P* = 0.01), and butyrate (r = 0.55, *P* = 0.04)
			- Group with obesity had higher carbohydrate fermentation by gut microbiome (*P* = 0.018).
Wan *et al.*, 2020 [29]	- Young adults (*n* = 163) - BMI classification comparison (underweight ≤18.5, normal 18.5–24, and overweight 24–28)	- Microbiome (16S rRNA) - Stool metabolites (SCFAs and intermediates of tricarboxylic acid cycle)	- No difference in microbiome composition
			- Higher α-diversity and richness in normal weight group than overweigh group (*P <* 0.01)
			- Overweight group had higher succinic acid (*P <* 0.001) and adipic acid (*P <* 0.05) than underweight and normal weigh, and had higher fumaric acid (*P <* 0.05), malic acid (*P <* 0.001), and propionate acid (*P <* 0.001) than normal weight
Yun *et al.*, 2017 [30]	- People healthy or with type 2 diabetes and/or hypertension (*n* = 1274) - BMI classification (normal 18.5–23, overweight 23–25), and obese ≥ 25)	- Microbiome (16S rRNA and genetic functional capacity)	- Group with obesity had lower α-diversity than those with overweight and normal weight (*P <* 0.01)
			- Higher expression of genes related to energy, and cofactors and vitamins metabolism in the group with obesity (*P <* 0.001)
			- Higher expression of genes related to lipid metabolism in the normal weight group (*P <* 0.001)
			- Regression analysis comparing overweight with normal weight showed: *Cyanobacteria* (B = 0.618, *P <* 0.001), *Desulfovibrio* (B = 0.435, *P <* 0.001), Paraprevotellaceae (B = 0.360, *P <* 0.001), *Acidaminococcus* (B = 0.331, *P* = 0.002), and *Eggerthella* (B= -0.155, *P* = 0.005)
			- Regression analysis comparing obesity with normal weight showed: *Acidaminococcus* (B = 0.498, *P <* 0.001), Paraprevotellaceae (B = 0.463, *P <* 0.001), *Megasphaera* (B = 0.443, *P <* 0.001), and *Eggerthella* (B = -0.162, *P* = 0.003)

Abbreviations: BMI: Body mass index; F/B ratio: Firmicutes to Bacteroidetes ratio; REE: Resting energy expenditure; SCFA: Short-chain fatty acid; RER: respiratory exchange ratio; TEE: Total energy expenditure (kcal/d); TREE: Total relative energy expenditure (kcal/kg/d).

**TABLE 2:** Summary of results from intervention studies assessing energy metabolism and the gut microbiome.

Reference	Population and time	Intervention	Assessments	Main outcomes
**Parallel arms randomized controlled trials**				
Bendtsen *et al.,* 2018 [31]	- People with overweight or obesity (*n* = 52) - 24 wk	- Energy restriction with low dairy diet (<600 mg of calcium/d) or high dairy diet (∼1500 mg of calcium/d, ∼1200 mg from dairy products)	- REE and RER (metabolic cart) - Microbiome (16S rRNA)	- Reduced weight and fat mass, and REE in both groups
				- RER decreased in high dairy (-0.02, *P <* 0.05) and tended to increase in low dairy (0.02, *P <* 0.10) (adjusted group effect *P* = 0.006)
				- Relative abundance of *Papillibacter* in wk 24 correlated with fat mass loss (r_s_ = 0.61, *P* = 0.017)
				- Low dairy decreased *Veillonella* (*P* = 0.014)
				- High dairy had no significant taxonomic change
Canfora *et al.*, 2017 [32]	- People with overweight or obesity with prediabetes (*n* = 44) - 12 wk	- Fiber (galactooligosaccharides, 5g 3x/d) compared to placebo	- REE and RER (metabolic cart) - Plasma and fecal SCFAs - Microbiome (16S rRNA)	- No alterations in plasma or fecal SCFAs, REE and substrate oxidation (RER)
				- Fiber increased *Bifidobacterium* (*P* = 0.009), *Prevotella oralis* (*P* = 0.010), *Prevotella melaninogenica* (*P* = 0.008), *Bacteroides stercoris* (*P* = 0.011) and *Sutterella wadsworthia* (*P* = 0.002)
Karl *et al.*, 2017 [33]	- Soldiers under intense training (*n* = 73) - 4 d	- Control diet - Protein-supplemented diet (+ 4 whey protein-based snack bars) - Carbohydrate-supplemented diet (+ 4 carbohydrate-based snack bars)	- TEE (DLW) - Microbiome (16S rRNA) - Plasma and fecal metabolomics - Intestinal permeability (sucralose excretion)	- No differences between diet groups for any of the following outcomes observed: weight loss, higher TEE, increase in intestinal permeability, increase in α-diversity, increased F/B ratio.
				- Decrease in metabolites of amino acid, fatty acid, carbohydrate, and energy metabolism
				- Amino acid and nucleotide metabolites were predictive for microbiome composition
				- Intestinal permeability correlated with pretraining *Actinobacteria* (r_s_ = -0.53, *P* = 0.01) Proteobacteria (r_s_ = 0.64, *P* = 0.002) and *Sutterella* (r_s_ = 0.68, *P* = 0.001) relative abundance
Karl *et al.*, 2017 [34]	- Men and postmenopausal women (40–65 y with BMI 20–35, *n* = 81) - 6 wk	- Whole grains-based diet (16 ± 0.8 g of fiber) compared to refined grains-based diet (8 ± 0.4 g of fiber)	- REE (metabolic cart) - Microbiome (16S rRNA) - Energy and SCFAs in stool	- Whole grains increased REE and stool energy content whereas refined–decreased (*P* = 0.04 and *P <* 0.001)
				- Refined grains decreased propionate (*P* = 0.05) and acetate (*P* = 0.02) compared to whole grains
				- Whole grains tended to decrease *Enterobacteriaceae* and to increase *Lachnospira* and *Roseburia.* No significant associations.
Most *et al.*, 2017 [35]	- People with overweight or obesity (*n* = 37) - 12 wk	- Polyphenols (epigallocatechin-3-gallate 282 mg/d and resveratrol 80 mg/d) compared to placebo	- TEE and fat oxidation (metabolic cart) - Mitochondrial oxidative capacity - Microbiome (RT-PCR)	- Men had higher Bacteroidetes than women at baseline (*P <* 0.01)
				- Polyphenols decreased Bacteroidetes compared to placebo in men (*P* = 0.05) but not in women (*P* = 0.15)
				- Polyphenols increased fat oxidation (fasting *P* = 0.03, postprandial *P* = 0.02) and mitochondrial oxidative capacity (*P* = 0.01)
				- Baseline Bacteroidetes correlated with postprandial fat oxidation in men (r = 0.855, *P* = 0.01)
Müller *et al.*, 2020 [36]	- People with normal weight or overweight (*n* = 48) - 12 wk	- Fiber (arabinoxylan-oligosaccharides, 15 g/d) compared to placebo	- Substrate oxidation and TEE (metabolic cart) - Microbiome (16S rRNA) - Fecal and plasma SCFAs	- Fiber tended to increase fat oxidation (*P* = 0.073) whereas placebo tended to decrease it (*P* = 0.089) (group effect *P* = 0.008).
				- No effect on TEE and carbohydrate oxidation
				- Fiber reduced α-diversity (*P <* 0.001)
				- Fiber changed gut microbiome composition (*P* = 0.05), exemplified by increased abundance of *Bifidobacterium*, *Akkermansia*, *Prevotellaceae* NK3831 group *Lactobacillus*, and decreased abundance of *Blautia*, *Eubacterium hallii*, *Coriabacteriaceau* and *Dorea*
				- No differences between fiber and placebo in fecal and plasma SCFAs
Yu *et al.*, 2020 [37]	- People with obesity (*n* = 22) - 12 wk (6 wk receiving intervention + 6 wk follow-up)	- FMT from healthy donors with normal weight to those with obesity, compared to placebo	- REE (metabolic cart) - Microbiome (16S rRNA and metagenomic)	- No change in REE, weight, and body composition
				- Compared to placebo, the microbiome profile of FMT recipients approached the microbiome of donors (*P <* 0.001) and were less similar to their own baseline microbiome (*P <* 0.05). Engraftment persisted during the 12 wk.
				- No significant correlations
**Crossover randomized controlled trials**				
Basolo *et al.*, 2020 [38]	- Healthy adults (*n* = 25) - 17 d (4 d run-in + 3 d of intervention + 3 d wash-out + 3 d intervention + 4 d wash-out)	- Overfeeding (OF, 150% of weight-maintaining diet, WMD) or underfeeding (UF, 50% of WMD) with provided diets	- Energy in stool and urine - Microbiome (metagenomic) - Plasma SCFAs and bile acids	- OF excreted more absolute calories in stool (*P <* 0.001) and urine
				- UF excreted more calories as a % of ingested calories in stool (*P <* 0.001) and urine (*P <* 0.001)
				- Plasma butyrate increased in OF (*P <* 0.001) and decreased in UF (*P* = 0.007)
				- UF decreased plasma deoxycholic acid (*P* = 0.012)
				- UF increased total colonization (*P* = 0.005) and α-diversity (*P* = 0.004)
				- UF increased Verrucomicrobia (p=0.01)
Jumpertz *et al.*, 2011 [39]	- Individuals with normal weight or obesity (*n* = 21) - 15 d (3 d run-in + 3 d intervention + 3 d wash-out +3 d intervention + 3 d wash-out)	- Run-in and wash-out diet with WMD - Overfeeding (OF) with either 2400 kcal or 3400 kcal per d	- Energy in stool, and urine - Microbiome (16S rRNA)	- Calories consumed in 3400 kcal intervention correlated with relative abundance of Firmicutes (r = 0.47, *P* = 0.04) and Bacteroidetes (r = -0.47, *P* = 0.04)
				- Changes in stool calories (% of ingested calories) in OF was correlated with change in relative abundance of Firmicutes (r = -0.50, *P* = 0.02) and Bacteroidetes (r = 0.52, *P* = 0.01)
				- In subjects with normal weight, there was decrease in energy absorption (*P <* 0.05)
Kaczmarek *et al.*, 2019 [40]	- Healthy adults (*n* = 18) - 60 d (18 d intervention + 24 d wash-out + 18 d intervention)	- WMD with no *Brassica* diet (control) or same diet with broccoli (200 g + 20 g of raw daikon radish as a source of myrosinase to hydrolyze the glucosinolates)	- Plasma metabolites - Microbiome and funtional potential (16S rRNA)	- Broccoli consumption reduced F/B ratio
				- No differences in α-diversity, β-diversity was smaller in broccoli than in control (*P* = 0.03)
				- Broccoli increased Bacteroidetes (*P* = 0.03), reduced Firmicutes (*P* = 0.05), and, thus, F/B ratio (*P* = 0.01)
				- People with BMI <26 kg/m^2^ increased plasma metabolites, and BMI >26 kg/m^2^ decreased it
				- People with BMI <26 kg/m^2^ decreased F/B ratio (*P* = 0.04) in broccoli.
				- In broccoli, the peak in plasma glucosinolate metabolites was correlated with change in Bacteroidetes (r = 0.69, *P* = 0.04) and change in Firmicutes (r = -0.66, *P* = 0.05).
				- Broccoli had increased pathways related to endocrine system (*P* = 0.05), transport and catabolism (*P* = 0.04), and energy metabolism (*P* = 0.01), and decreased membrane transport (*P* = 0.03) compared to control.
Karusheva *et al.*, 2019 [41]	- Type 2 diabetes patients (*n* = 12) - 28 d (7 d run-in + 7 d intervention + 7 d wash-out + 7 d intervention)	- Intervention: diet with low branched-chain amino acids (<60% BCAA-) or with all essential amino acids (BCAA+)	- TEE (estimated) - Mitochondrial oxidative capacity - Microbiome (metabolomic)	- No difference in TEE and skeletal muscle mitochondrial oxidative capacity
				- BCAA- increased adipose tissue mitochondrial efficiency (*P <* 0.05)
				- BCAA+ increased indicators of β-oxidation in adipose tissue (*P <* 0.05)
				- BCAA- had reduced Firmicutes (*P <* 0.05) and increased Bacteroidetes (*P <* 0.05) compared to BCAA+
Papadopoulou *et al.*, 2020 [42]	- Healthy adults (*n* = 17) - 4 wk (1-wk intervention + 2 wk wash-out + 1-wk intervention)	- Intervention: 60% of diet consumed either at lunch or dinner	- Stool energy and SCFAs - Microbiome (16S rRNA)	- No difference in stool characteristics, energy, and SCFAs
				- Microbiome clustered by enterotype, not meal timing
				- When participants had large lunch there was higher concentration of *E. coli* (*P* = 0.03)
**Single-arm interventions**				
Kelder *et al.*, 2014 [43]	- Healthy men (*n* = 10) - 4 wk	- High-fat high-caloric diet (HFHC) was provided to all participants	- REE (metabolic cart) - Fecal SCFAs - Microbiome (16S rRNA)	- HFHC increased body weight (*P <* 0.001), carbohydrate oxidation (*P <* 0.001), and REE (*P <* 0.05)
				- HFHC did not change overall microbiome composition
				- Correlation network show that carbohydrate oxidation had a positive correlation with taxa in Firmicutes phylum and negative correlation with taxa in Bacteroidetes phylum (r and p values not shown)
				- Multiple SCFAs correlated with Porphyromonadaceae and Sutterellaceae
				- REE had a positive correlation with P/B ratio and *Prevotellaceae*
Ott et al., 2018 (44)	- Healthy males with normal weight (*n* = 24) - 4 wk (7 d run-in + 7 d intervention + 14 d wash-out	- Run-in and wash-out participant’s usual WMD - Intervention HFHC	- REE (metabolic cart) - Gut permeability - Microbiome (metagenomic)	- No difference in REE
				- No consistent difference in gut permeability
				- No difference in α-diversity
				- HFHC decreased *Bacteroidaceae* and increased *Betaproteobacteria*

Abbreviations: BCAA: Branched-chain amino acids; BMI: Body mass index; DLW: Doubly labeled water; F/B ratio: Firmicutes to Bacteroidetes ratio; FMT: Fecal microbiome transplantation; HFHC: high-fat high-caloric diet; OF: Overfeeding; REE: Resting energy expenditure; P/B ratio: *Prevotella* to *Bacteroides* ratio; RER: respiratory exchange ratio; RT-PCR: Reverse transcription polymerase chain reaction; SCFA: Short-chain fatty acid; TEE: Total energy expenditure; UF: Underfeeding; WMD: weight-maintaining diet.

## An overview of special considerations regarding the assessment of energy metabolism and gut microbiome composition and functions

**FIGURE 2:**
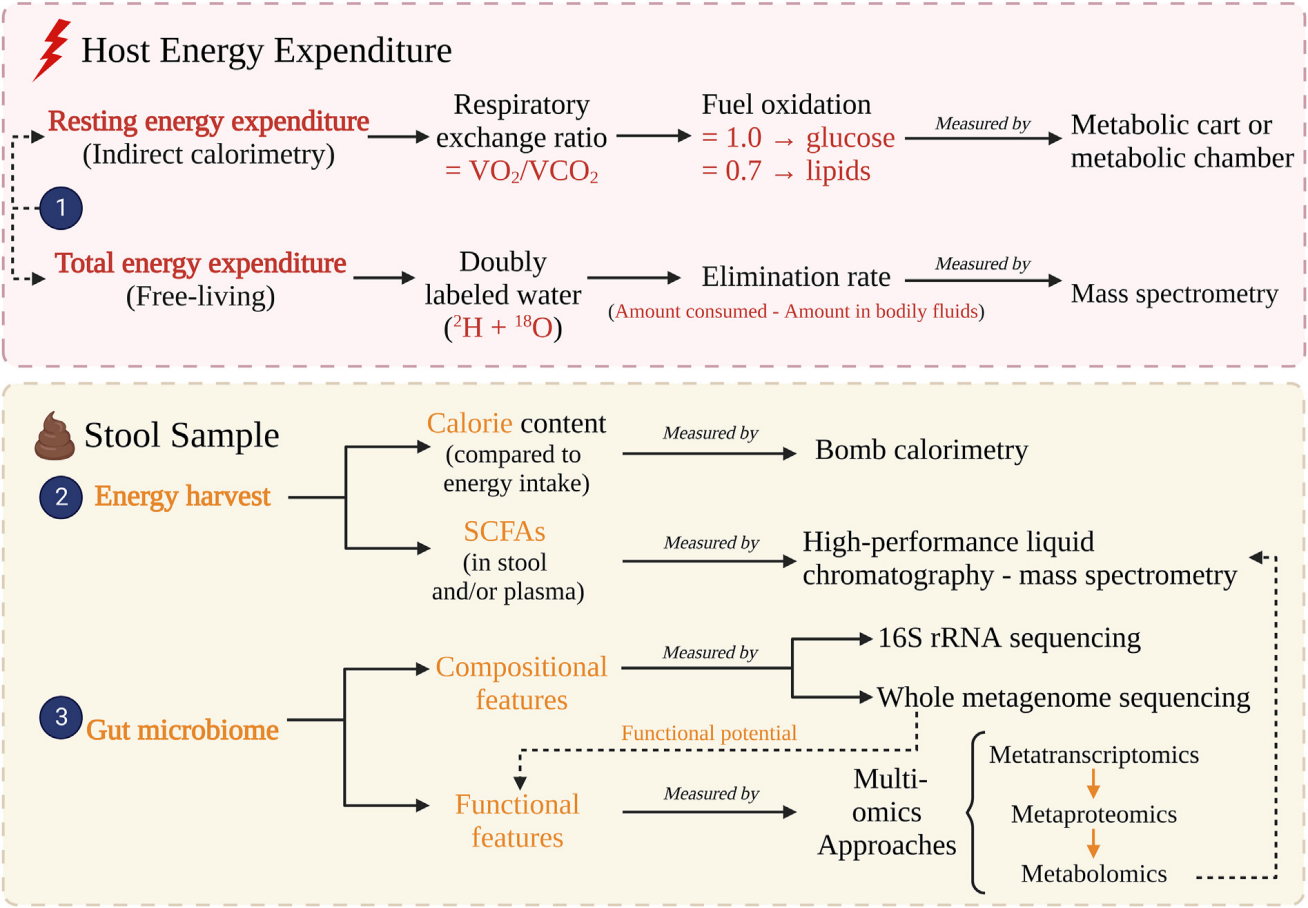
**Methods for assessing energy metabolism and gut microbiome**. 1) Host energy expenditure can be assessed by indirect calorimetry or doubly labeled water; 2) In stool samples, the caloric content and short-chain fatty acid (SCFA) can be analyzed to infer energy harvest by the gut microbiome; 3) Gut microbiome composition and metabolic functions can be assessed by 16S rRNA sequencing or metagenomic sequencing, which data can be used to infer functional potential, gut microbiome functional features can also be assessed with multi-omics approach.

Prior to discussing the selected literature, it is important to summarize the methodologies currently used to assess energy metabolism and the gut microbiome in human studies. Three main methods, summarized in Figure 2, have been used to provide insight into the impact of gut microbiome on host energy metabolism: 1) energy expenditure assessment of the host; 2) comparison of energy intake to energy in stool; and 3) assessment of gut microbiome composition, functions encoded in microbial genomes (assessed by metagenomics), and metabolites (including SCFAs).

The net outcome of host energy metabolism can be evaluated by measuring energy expenditure, primarily REE. In oxidative phosphorylation, oxygen (O_2_) is used to transfer energy from fuel (e.g., glucose and fatty acids) to adenosine triphosphate. The by-products of this process are carbon dioxide (CO_2_) and water (H_2_O) [45]. In indirect calorimetry methods, energy expenditure is estimated based on this gas exchange (i.e., volume of inspired O_2_ and volume of expired CO_2_). The indirect calorimetry method can also be used to estimate substrate oxidation by comparing the respiratory exchange ratio (RER)—the ratio of the volumes of CO_2_ and O_2_—to the standard RER values for carbohydrate, lipid, and protein substrates [45, 46]. Methods to analyze energy expenditure by indirect calorimetry include metabolic chambers and metabolic carts. Metabolic chambers are airtight whole-body rooms where the volume of gas exchange is calculated based on the difference between the volumes in the air introduced versus the air withdrawn from the chamber. There are less than 50 whole-body units in the world that quantify total energy expenditure. As such, metabolic carts are more commonly used due to their lower cost and greater availability compared to metabolic chambers. Metabolic carts measure REE using ventilated hoods or facemasks that capture gas volumes either breath-by-breath or by an open circuit flow [46]. Doubly labeled water (DLW) is an additional method that can be used to measure TEE in free living conditions. In this method, deuterium hydrogen (^2^H) and oxygen (^18^O) water is ingested, and daily disappearance is analyzed in urine by mass spectrometry. Expired CO_2_ volume is estimated, considering ^2^H is only excreted as water, whereas ^18^O is excreted both as water and CO_2_ [46, 47].

Combined energy harvested by the host and gut microbiome can be estimated by comparing caloric intake with caloric content of stool. Bomb calorimetry is the gold standard for measuring stool energy content [48]. In the context of the microbiome, this is especially important for fiber as it is not digested by human digestive enzymes; however, some fibers can be fermented by the gut microbiome. Importantly, bomb calorimetry may overestimate harvestable caloric content of stool as not all fiber residues can be fermented by the gut microbiota (e.g., cellulose is poorly fermented by the human gut microbiome) [49]. Alterations in fecal SCFAs can be correlated with gut microbiome changes [50], but one must consider that only an estimated 5% of SCFAs produced in the gut escape absorption and are excreted in feces, and there are no validated formulas by which energy generation from SCFAs can be determined. Furthermore, different conditions can affect the amount of fat in stool, such as calcium intake [51] and diseases (e.g., pancreatitis and intestinal malabsorption) [52]. Plasma SCFAs may give further insight into the number of SCFAs absorbed that could exert systemic effects [9]. SCFA content is mainly analyzed using gas chromatography and high performance liquid chromatography, often coupled with mass spectrometry [50].

To characterize the gut microbiome, most studies utilize 16S rRNA gene amplicon sequencing or metagenomic sequencing. Although gene amplicon sequencing provides taxonomic information, it provides no direct information about microbial genetic functions [53]. In contrast, metagenomic sequencing can be used to infer both the taxonomic identity and functional potential of the microbial genes present in a sample. However, microbes have various metabolic pathways that can be activated under different circumstances; thus, this approach may not correspond to the actual functions being performed by the microbiome [54, 55]. Metatranscriptomic, metaproteomic, and metabolomic approaches provide a better picture of actual microbial activity in the gut environment. Unfortunately, there remain significant limitations in our ability to accurately assess microbiome functionality from fecal samples, and studies on energy metabolism testing multiple “omics” approaches in humans are still scarce.

## Gut microbiome and energy metabolism in diverse nutritional statuses

Among included studies, there were cross-sectional observational studies reporting associations between gut microbiome and nutritional status [[25], [26], [27], [28], [29], [30]]. Several studies compared microbiota composition and function in adults with different BMI classifications and used at least one of the methods described above to assess its possible relationship with energy metabolism.

Two studies compared the microbiomes in people of different BMI categories according to Asian classifications [29, 30]. In both studies, dietary information was collected and used for adjusting statistical analyses. Both studies found no differences in overall microbiota composition among BMI classes; however, α-diversity was inversely correlated with BMI [29, 30]. Functional metagenomic analysis showed an over-representation of genes related to microbial ‘Energy Metabolism’ and ‘Metabolism of Cofactors and Vitamins’ in participants with overweight (BMI 23 – 25 kg/m^2^) and obesity (BMI ≥25 kg/m^2^) compared with normal weight (BMI 18.5 – 23 kg/m^2^) according to Asian-Pacific BMI criteria [30]. Microbial pathways related to lipid metabolism, excretory and endocrine systems, and xenobiotics biodegradation were depleted in the group with obesity. Decreases in carbohydrate, pyruvate, and amino acid metabolism were also detected in participants with obesity [30]. In the study by Wan et al., selected intestinal metabolites (succinic, fumaric, malic, propionic, and adipic acids) were higher in participants with overweight (using the Chinese obesity criteria, BMI 24–28 kg/m^2^) compared with those with underweight (BMI <18.5 kg/m^2^) and normal weight (BMI 18.5–24 kg/m^2^) [29]. Succinate, fumarate, and malate are intermediates in the tricarboxylic acid cycle, an important cellular cycle for energy production. The higher propionic acid in people with overweight is in line with previous findings that SCFAs are higher in subjects with obesity [56], although no differences were found for butyric acid, and acetic acid was not analyzed [29].

In these same 2 studies, correlations were found between specific taxa and BMI classification. The relative abundance of *Veillonellaceae* had a weak positive correlation (r < 0.4) with body weight [29]. In a multiple regression model, there was a strong positive effect of *Cyanobacteria* in overweight (BMI 23–25 kg/m^2^) compared with normal weight (BMI 18.5–23 kg/m^2^), a moderate effect for *Desulfovibrio*, *Paraprevotellaceae*, and *Acidaminococcus*, and a weak effect for *Eggerthella*. In the group with obesity (BMI ≥25 kg/m^2^), a moderate effect for *Acidaminococcus*, *Paraprevotellaceae*, and *Megaspharea*, and a weak effect for *Eggerthella* were observed, compared with those with normal weight [30]. In the study by Yun et al*.,* the total caloric intake was higher in the group with obesity, without differences in macronutrient distribution [30]. The higher caloric intake alone may contribute to the nutritional status observed, regardless of the influence of the microbiome on energy metabolism, and it might also explain differences in microbiota composition, as diet influences the microbiota. This study used a food frequency questionnaire designed for the Korean population [30]. Notably, food frequency questionnaires have low accuracy with recall bias; other methods for analyzing food intake, such as diet records, could have improved the assessment of energy intake [57]. On the other hand, Wan et al. observed no statistical difference in total energy intake between BMI classes; however, those in the overweight category had higher fat and lower carbohydrate and fiber intakes, and food quality can impact thermogenesis [29]. In this case, caloric intake alone did not explain differences in BMI, suggesting that other factors, such as physical activity or the microbiome, may have contributed [29].

The study by Goffredo et al*.* [28] compared children and adolescents according to BMI percentiles and reported associations between body fat partitioning (assessed by magnetic resonance imaging) and gut microbiome taxa. They found that Firmicutes to Bacteroidetes (F/B) ratio and the relative abundance of Bacteroidetes and Actinobacteria were associated with BMI, visceral and subcutaneous adipose tissues, and hepatic fat [28]. Inconsistencies with the F/B ratio have been reported in relation to obesity, and this will be contemplated in the discussion section. F/B ratio and Bacteroidetes were correlated with acetate fecal concentration. Adipose tissue compartments were also correlated with fecal acetate, propionate, and butyrate. Hepatic lipogenesis had a moderate association with acetate and butyrate. The gut microbiome of the group with obesity showed a higher carbohydrate fermentation than the group with normal wiehgt, indicating higher energy harvest [28].

The energy harvesting capacity of one’s gut microbiome may contribute to body weight phenotypes. Boekhorst et al. identified 3 enterotypes in people with overweight or obesity (BMI 25–35 kg/m^2^): *Bacteroides* (B-type), *Prevotella* (P-type), and *Ruminococcaceae* (R-type) [26]. Such enterotypes showed different energy harvesting capacities, as indicated by stool energy density, which was lower in B-type than R-type. Stool energy density was positively correlated with transit time, which was shorter in B-type than in R-type. Transit time was also positively correlated with stool SCFAs. The R-type had higher levels of stool branched SCFAs (isobutyrate, 2-methylbutyrate, and isovalerate) than B-type. P-type results were in between B-type and R-type, but P-type had higher levels of stool valerate and caproate than B-type (26). The authors suggested that B-type had higher energy harvesting capacity, with potential for higher SCFAs production and absorption. Furthermore, R-type had higher levels of urine proteolytic metabolites than B-type and P-type [26], indicating higher protein fermentation by this enterotype.

In a study by Ghosh et al., nutritional status of children from India was assessed based on z-scores (i.e., height for age, weight for age, and weight for height). Children were then categorized as apparently healthy, borderline malnourished, or severely malnourished based on their cumulative nutritional status (i.e., the sum of the 3 z-scores) [27]. Network analysis was used to identify co-occurring genera, which were then used to divide participants into 4 groups based on abundance patterns of microbial taxa—the 4 groups were arbitrarily named G1 – G4. Compared with malnourished children, children with good nutritional status presented a higher abundance of taxa from G1, which included the SCFA-producing genera *Roseburia*, *Faecalibacterium*, and *Butyrivibrio*. Malnourished children presented higher abundance of taxa belonging to G4, which included *Escherichia*, *Streptococcus*, *Shigella*, *Enterobacter*, and *Veillonella*. Details on type of delivery and neonatal feeding information were not included, despite their potential impact on gut microbiome composition [58]. There was no significant correlation between nutritional status and G2 and G3. Children with good nutritional status had an over-representation of specific microbial categories of functional potential related to nutrient uptake and metabolism (e.g., secondary metabolite biosynthesis, transport, and catabolism); energy production and conversion; amino acid transport and metabolism; and carbohydrate transport and metabolism [27]. This suggests a more effective nutrient and energy utilization by the gut microbiome, which could increase the availability of nutrients to the host. Moreover, functional categories associated with virulence and bacterial pathogenesis were over-represented in children with poor nutritional status, including intracellular trafficking; secretion and vesicular transport; cell motility; and inorganic ion transport and metabolism [27]. Pathogenic bacteria need to secure nutrients and metals such as iron and zinc from the host to grow and avoid immune clearance [59, 60]. This may explain the relationship between functions associated with pathogenicity and poor nutritional status.

An observational study by Bielik et al. compared normal weight athletes (BMI of 18.5–24.9 kg/m^2^) who were in positive energy balance (case group) with those with a caloric intake that met their energy requirement (control group) [25]. No differences in REE and TEE between the 2 groups were reported. Athletes in the case group had higher macronutrient and total energy intake and lower fiber intake compared with athletes in the control group. Thus, the average difference between energy intake and expenditure was significantly higher in the case group. However, body weight and BMI were significantly lower in the case group [25], which was unexpected as positive energy balance is associated with higher BMIs. Other factors were likely influencing energy metabolism in the case group, potentially including the gut microbiome. Athletes in the case group presented with lower levels of Gammaproteobacteria, *Shewanella*, *Xanthomonas*, Alteromonadales, and *Coriobacteriaceae* and higher levels of *Roseburia* spp. and *Barnesiella* spp. compared with athletes in the control group. The authors reported moderate correlations between select bacterial taxa and energy metabolism parameters. For instance, *Coriobacteriaceae* was positively correlated with BMI and negatively correlated with carbohydrate intake. *Roseburia* spp. were positively correlated with body fat, total energy intake, TEE/body weight; kcal/kg/d), resting oxygen consumption, and carbohydrate intake, and negatively correlated with RER. Alteromonadales were negatively correlated with resting oxygen consumption [25]. The muscle damage and lactate production that occurs during strenuous physical activity increases the energetic demand, increasing resting oxygen consumption. However, the increase in oxygen consumption is not equal to the oxygen necessary to metabolize lactate [61]; thus, the correlations suggest a possible contribution of the microbiome to oxygen consumption. These correlations were found in both groups and indicated that gut microbiome metabolism might be important in modulating energy metabolism; however, mechanisms were not elucidated.

## Alterations in dietary patterns impact gut microbiome and energy metabolism

Four studies [38, 39, 43, 44] investigated how alterations in diet patterns can influence gut microbiome composition and functions in relation to alterations in host energy metabolism. Basolo et al. conducted a crossover feeding trial to compare gut microbiome and host metabolic alterations resulting from overfeeding and underfeeding while maintaining the same diet composition [38]. They observed that underfeeding significantly increased total microbial load and α-diversity compared with overfeeding. Microbial community structure and interindividual differences were maintained from baseline to the end of both interventions. The phylum Verrucomicrobia, *Akkermansia muciniphila,* and *Alistipes* spp. increased after underfeeding, whereas *Lachnospiraceae* spp. decreased. Absolute calories lost in stool and urine were higher during overfeeding. However, underfed subjects had a higher percentage of calories lost in stool relative to calories ingested, which may be related to the faster transit time observed in this group with reduced fiber fermentation or endogenous energy loss in stool, which was not tested. They observed that underfeeding decreased plasma deoxycholic acid, a secondary bile acid that increases gut permeability. Plasma SCFA content, especially butyrate, was decreased during underfeeding [38]. This may indicate reduced substrate availability. Hence, in addition to the lower caloric and nutrient intakes caused by underfeeding, higher energy loss in stool and lower energy harvesting by the microbiome may have increased the caloric deficit of the host. The study also provides potential explanations for microbiome alterations observed in obesity, as overfeeding is associated with similar changes seen in individuals with obesity, which, on average, eat more than individuals with normal weight.

Similarly, Jumpertz et al. investigated how changing from a weight-maintaining diet to a higher-calorie diet altered the gut microbiome and energy excretion in individuals with or without obesity [39]. Overfeeding was associated with an increase in Firmicutes, a decrease in Bacteroidetes, and, thus, an increased F/B ratio [39]. In individuals without obesity, nutrient absorption was positively associated with Firmicutes and negatively associated with Bacteroidetes; phylum-level changes were associated with calories in stools. The finding that overfeeding increased the F/B ratio suggests that changes in this ratio and obesity are both consequences of dietary changes and that a cause-effect relationship between this particular change in the gut microbiome and obesity is unlikely. Similarly to the study by Basolo et al. [38], Jumpertz et al. observed a decrease in stool energy loss in overfeeding compared with the weight-maintaining diet that was significant in individuals without obesity but not in those with obesity [39].

Two studies analyzed the impact over time of a high-fat, high-calorie (HFHC) diet in healthy males [43, 44]. The HFHC diet did not affect the F/B ratio [43] or α-diversity [44]. Both studies showed that interindividual differences prevailed over HFHC-induced differences [43, 44]. The bacterial families *Porphyromonadaceae* and *Sutterellaceae* showed strong positive correlations with multiple SCFAs [43]. Kelder et al. observed that the HFHC diet increased body weight and visceral fat area [43]. They also observed an increase in carbohydrate oxidation, energy expenditure, and REE [43]; however, this was likely insufficient to compensate for the higher caloric intake. Host carbohydrate oxidation was positively correlated with all taxonomic groups within the Firmicutes phylum and negatively correlated with all taxonomic groups within the Bacteroidetes phylum. Fat oxidation and RER had a strong negative correlation with the genus *Clostridium*, which belongs to the phylum Firmicutes [43]. A strong positive correlation was found between REE and the *Prevotella* to *Bacteroides* (P/B) ratio and *Prevotellaceae* family [43]. Two enterotypes characterized by the predominance of *Prevotella* or *Bacteroidetes* have been identified and will be acknowledged in the discussion. Ott et al. observed increases in body weight and fat mass during the HFHC diet, but no differences in REE, waist circumference, hip circumference, or lean mass were reported [44]. However, neither of the studies confirmed if compositional changes in the gut microbiome were relevant to the alterations in body weight, body composition, and energy metabolism. Notably, although microbial function may be altered by dietary changes, this was not assessed in these studies.

The increase in calorie intake may increase the number of nutrients available for the gut microbiome [62], as observed by an increase in absolute energy in stool in overfeeding studies [38, 39]. Different microbes have different metabolic capacity for each macronutrient. In the case of HFHC diets, the increase in fat available may have favored strains capable of metabolizing lipids and increased mucin use due to the lack of dietary fiber [62]. Furthermore, increased fat intake may have increased bile production, which, in turn, affects gut microbiome composition due to the antimicrobial properties of bile acids, although some microbes can metabolize primary bile acids into secondary bile acids [63]. Correlations between microbial taxa with substrate oxidation and energy expenditure [43] indicate that the gut microbiome may contribute to the increase in weight and fat mass related to HFHC diets, although the degree of its contribution is still unknown.

## Impact of dietary fiber on the gut microbiome and energy metabolism

Several authors have tested the effects of fiber supplements such as galactooligosaccharides (GOS) [32] and arabinoxylan-oligosaccharides (AXOS) [36] on gut microbiome and energy metabolism parameters. Microbiome α-diversity was unchanged by GOS [32] but was reduced by AXOS compared with placebo [36]. These oligosaccharides increased the relative abundance of *Bifidobacterium* taxa; however, there were no significant changes in fecal SCFAs [32, 36]. GOS did not affect REE, RER, fat oxidation, and carbohydrate oxidation [32]. AXOS tended to increase postprandial fat oxidation but did not change energy expenditure, RER, and carbohydrate oxidation [36]. There were no significant alterations in BMI, body composition, or dietary intake (including fiber intake besides the fiber supplement) compared to placebo [32, 36]. Although changes in microbiome were observed, both fiber supplements were ineffective at modulating energy metabolism.

Isolated fibers such as those used in the studies described above may exert different effects on the gut microbiome and host energy metabolism than fiber present in whole foods. Such intrinsic fiber may interact with other nutrients in food, such as lipids, proteins, and bioactive compounds, such as phytochemicals. Considering this, Karl et al. compared a diet based on fiber-rich whole grains with another based on fiber-poor refined grains [34]. Plasma alkylresorcinol, a biomarker of whole grain intake, increased in the whole grain group and was unchanged in the refined grain group, indicating adherence to the dietary intervention. Whole grain consumption tended to decrease proinflammatory *Enterobacteriaceae* and to increase butyrate-producing *Lachnospira* and *Roseburia* compared with refined grain consumption [34]. Stool weight increased in the whole grain group, leading to higher total stool energy content, though stool energy density did not differ between groups. This was accompanied by an increase in REE, leading to a higher energy output compared with the refined grain group. There was a decrease in stool propionate in both groups, whereas stool acetate decreased only in the refined grain group. This was reflected in a decreased concentration of total SCFAs in the stool of participants in the refined grain group compared with the whole grain group [34]. Changes in SCFAs suggest that reduced fiber content in the refined grains diet limited substrate availability for gut microbes, therefore, reducing fiber fermentation and SCFA production. The reduction in acetate in the refined grain group may decrease lipogenesis [10] and browning of white adipose tissue [11]. Changes in stool weight and energy content had moderate positive correlation with changes in plasma alkylresorcinol. Changes in REE, stool weight, and stool energy content were not associated with changes in the relative abundance of any taxon. This indicates that observed alterations in energy metabolism were likely caused by whole grain intake independent of changes in fecal microbiome composition [34].

Another way to increase dietary fiber intake is through increased vegetable consumption. In a crossover trial, Kaczmarek et al. compared a control diet low in vegetables from the *Brassica* genus (e.g., broccoli, cauliflower, Brussels sprouts, cabbage, etc.) to the same diet with broccoli (200 g) added [40]. They found no significant differences in relative bacterial abundance or α-diversity between treatments. Average interindividual β-diversity was reduced during broccoli consumption, indicating that this treatment may cause bacterial communities to become more similar to one another. The broccoli intervention increased the relative abundance of Bacteroidetes and reduced the relative abundance of Firmicutes, thus, reducing the F/B ratio. When participants were divided according to BMI, those with lower BMI (<26 kg/m^2^) presented with a significant decrease in the F/B ratio. Overall, broccoli consumption upregulated pathways involved in endocrine system function, systemic transport, catabolism, and energy metabolism, whereas downregulating pathways involved in membrane transport [40]. Although fiber was probably a key factor in causing these alterations, broccoli has bioactive compounds that may also play a role in these findings, such as hydrolysable glucosinolates [64]. In the broccoli intervention, peak in plasma metabolites was strongly and positively correlated with change in Bacteroidetes and strongly and negatively correlated with change in Firmicutes [40]. Some gut microbes have been identified to hydrolyze glucosinolates into either bioactive isothiocyanates or inert nitriles [65]. Similarly, other dietary fiber sources such as cereals, fruits, and vegetables [66] have bioactive compounds, which may act synergistically with fiber and SCFAs affecting energy metabolism and gut microbiome.

## Gut microbiome and energy metabolism in other interventions

Fecal microbiome transplantation (FMT) is a method that could be used to evaluate the hypothesized causal role of the gut microbiome in modulating energy metabolism. Yu et al. performed FMT from donors with normal body weight to people with obesity [37]. They observed that the microbiomes of transplant recipients were more similar in composition to their paired donor and less similar to their own baseline sample compared to placebo recipients. All transplant recipients exhibited engraftment of donor-specific bacteria (amplicon sequence variants) within 3 wk of repeated FMT, which persisted for all 12 wk of the study, including 6 wk without additional FMT. However, there was high variability in the relative abundance of donor-specific bacteria in the microbiomes of transplant recipients. No significant change in REE pre- and post-FMT and no difference between groups were reported [37]. These findings indicate that although microbes from the donor microbiomes effectively colonized recipients’ gastrointestinal tract, altering microbiome composition alone was insufficient for improving host energy metabolism. A change in microbiome composition does not necessarily lead to changes in metabolic pathway activation [67], which could explain the lack of alterations in energy metabolism in this study [37]. A metabolomics approach to analyzing the microbiome could help shed light on the functions related to energy metabolism in FMT compared with the control.

Higher physical activity influences host energy metabolism, and some of these effects might be mediated through the gut microbiome. Karl et al. analyzed intense physical training combined with a control diet, a protein-supplemented diet, or a carbohydrate-supplemented diet [33]. All groups experienced weight loss and increased energy expenditure as assessed by DLW, with no differences among diet groups [33]. Energy intake was higher in the high carbohydrate group compared with the other 2 groups; however, this did not affect other outcomes. Fecal microbiome α-diversity and relative abundance of Firmicutes and Verrucomicrobia increased from baseline to post-training in all diet groups, whereas Bacteroidetes decreased, increasing the F/B ratio [33]. Furthermore, exercise training changed plasma metabolite profiles independently of diet, including changes in secondary bile acids: glycolithocolate sulfate, glycohyocholate, taurolithocholate 3-sulfate, and taurocholenate sulfate increased, whereas deoxycholate, ursodeoxycholate, and isoursodeoxycholate decreased. Several metabolites of amino acids, fatty acids, carbohydrates, and energy metabolism decreased in the stool samples of all groups. All groups had increased *p*-cresol after the exercise intervention, indicating microbial fermentation of protein regardless of diet. Prediction models identified that amino acid and nucleotide metabolites predicted microbiome composition. Those metabolites were positively associated with intestinal permeability, which increased during training. Intestinal permeability had a moderate inverse correlation with pretraining relative abundance of *Actinobacteria* and a moderate positive correlation with pretraining Proteobacteria and *Sutterella* relative abundance [33]. Overall, this study established associations between the physiological effects of physical activity on the host and changes in the microbiome, but whether the microbiome makes causal contributions to this effect was not established. The weight loss observed was likely the result of the intense training instead of dietary changes and gut microbiome modulation.

Phenolic compounds comprise a group of bioactive molecules with various chemical structures that could impact the gut microbiome [68] and energy metabolism [69]. A study by Most *et al.* showed that supplementation with polyphenols from wine (epigallocatechin-3-gallate and resveratrol) reduced Bacteroidetes in men, which had a higher relative abundance of Bacteroidetes at baseline compared to women [35]. Other taxa were unchanged in either sex. Supplementation increased fat oxidation and skeletal muscle mitochondrial oxidative capacity in both sexes. Baseline Bacteroidetes abundance had a strong positive correlation with the polyphenol-induced increase in postprandial fat oxidation in men but not in women [35]. They found no correlations between the reduction in Bacteroidetes and fat oxidation in men and no correlations between fat oxidation and microbial taxa in women. Thus, reduction in Bacteroidetes cannot explain the changes in fat oxidation.

The microbiome contributes to amino acid catabolism and production, and hence the concentration of circulating branched-chain amino acids (BCAAs) (70). A crossover trial compared the influence of an isocaloric diet low in BCAAs and a diet normal in BCAAs (complete set of BCAAs) on insulin sensitivity in adults with treated type 2 diabetes [41]. A lower abundance of Firmicutes and a higher abundance of Bacteroidetes were observed after the low BCAA intervention compared with the normal BCAA intervention, resulting in a decreased F/B ratio in the low BCAA group. Skeletal muscle oxidative capacity and TEE were similar after adoption of either diet. The low BCAA diet increased adipose tissue mitochondrial capacity and reduced β-oxidation compared to normal BCAA diet (41). Likewise, supplementing BCAA has been found to increase the F/B ratio. People with obesity and type 2 diabetes also had higher levels of circulating BCAA, and the microbiome of subjects with obesity synthesized more BCAA, with less BCAA breakdown [70].

Dairy intake may also influence the gut microbiome. Bendtsen et al. investigated energy restriction associated with either a low dairy or high dairy diet in subjects with no dairy allergies [31]. They found no significant change in diversity or overall microbiome composition in either dietary group. However, the relative abundance of the genus *Veillonella* was significantly decreased in the low dairy group, whereas *Papillibacter* was increased in the high dairy group. No differences between groups were observed for changes in fecal fat or energy excretion. There was a significant decrease in REE in the low dairy group from baseline; however, this was not statistically different from the change in the high dairy group. There was a significant difference in RER between groups, with a decrease in the high dairy group, indicating a shift toward fat oxidation and tendency to increase in the low dairy group. Energy, protein, carbohydrates, and total fat intakes were not different between groups; therefore, dietary factors did not contribute to differences in energy metabolism. There were differences in calcium, saturated fatty acids, and polyunsaturated fatty acids intakes, though the differences in RER remained significant when adjusted for fat intake. Reductions in energy intake, body weight, and hip and waist circumferences during the study were similar between the 2 groups. The relative abundance of *Papillibacter* showed a moderate positive correlation with total fat mass loss independent of diet group [31]. High dairy intake was associated with a change in fuel oxidation that was not explained by differences in nutrient intake. Although no major changes in gut microbiome were observed, microbial taxa may be involved in the modulation of energy metabolism, as exemplified by the correlation between *Papillibacter* and fat loss. However, mechanisms and causation cannot be inferred from these findings.

Finally, the effect of meal timing on the gut microbiome and host energy metabolism was tested in a crossover study comparing the consumption of a large lunch versus a large dinner, with 60% of energy requirements provided in each of those meals [42]. There were no changes in gut microbiome diversity after interventions and no differences between interventions. The time of main meal consumption did not influence total or taxon-specific bacterial fecal content except for *Escherichia coli*, which was significantly higher after the large lunch intervention. Stool characteristics, energy content, or SCFA content did not differ. There was an increase in body weight following the large dinner intervention only [42]. Minor changes in microbial composition associated with the timing of the main meal were not clearly associated with increased body weight.

In summary, the interventions described above were not associated with gut microbiome modulation to improve energy metabolism. To our knowledge, there is only one study for each type of intervention that assessed these outcomes (6 in total). Therefore, the paucity of experimental evidence in humans precludes any conclusion regarding the effect of how dietary and nondietary interventions affect energy metabolism in humans via microbiome modulation.

## Discussion

We reviewed studies that analyzed both the gut microbiome and energy metabolism in humans. In animal models, the gut microbiome has been clearly implicated as a causal factor influencing energy metabolism, weight gain, energy expenditure, and pathologic phenotypes related to obesity (14, 17), but confirming these findings in humans is challenging.

Several studies have attempted to characterize gut microbiome patterns—or enterotypes—that are associated with specific dietary patterns and nutritional status, especially obesity. Two enterotypes related to long-term dietary patterns have been identified: one dominated by *Prevotella* and another by *Bacteroides* [71]. The *Prevotella* enterotype was associated with diet patterns that contained high carbohydrates (total carbohydrates and simple sugars, but not fibers) intake, whereas *Bacteroides* was associated with diets high in animal protein (and all amino acids) and animal fat (saturated fatty acids) [71]. People with the *Prevotella* enterotype experienced greater weight loss during caloric restriction than people with the *Bacteroides* enterotype [72]. The *Bacteroides* enterotype is more common in Western populations, whereas the *Prevotella* enterotype is found more commonly in nonindustrialized populations, which typically consume a diet higher in fiber and lower in animal protein compared with industrialized populations [73]. It has also been observed that people with normal weight present with higher *Bacteroides* versus *Prevotella* relative abundance than those with obesity [17]. Furthermore, a higher P/B ratio is associated with greater weight and fat losses [74, 75].

Obesity has also been associated with a higher F/B ratio [17, 76]; however, studies have been inconsistent [77]. A meta-analysis found no significant association between this ratio and obesity, nor in the relative risk of obesity based on F/B ratio [77]. Additionally, the F/B ratio approach has been questioned for showing interpretative bias: since the first associations between this obesity and F/B ratio were observed, subsequent studies have focused on this ratio and disregarded other phyla and lower taxonomic levels [78]. Therefore, this association is insignificant and unlikely to contribute to energy balance and obesity development.

Different microbial taxa can affect inflammation, which, depending on the degree and extent, impact energy metabolism [13]. Inflammation induces leptin expression, which increases thermogenesis and energy expenditure and reduces food intake [12]. However, inflammation may also lead to behaviors to conserve energy, including fatigue and anhedonia [13]. Obesity is associated with low-grade chronic inflammation, likely due to the energy surplus that leads to adipose tissue expansion and hypoxia [12, 13]. However, in the context of obesity, resistance to this inflammatory effect of increased energy expenditure may occur mediated by leptin resistance [12]. Increased circulating LPS contributes to chronic low-grade inflammation, and it is considered obesogenic because it inhibits adaptative thermogenesis, reducing energy expenditure [14].

Of the studies included in this review, only 2 included analysis of bile acids in the stool or plasma [33, 38]. Although the metabolism of bile acids and their effect on signaling pathways are well understood in animal models [16], rodents produce predominantly the bile acids cholic acid and muricholic acids, whereas humans produce primarily chenodeoxycholic acid and cholic acid [63]; thus, those bile acids would be metabolized differently and would have varying levels of signaling pathways activation [16]. Therefore, it is important to analyze bile acid metabolism in humans and how it affects energy metabolism.

Most interventional studies hereby reviewed did not observe changes in body weight nor in body composition when both the gut microbiome and variables related to energy metabolism were altered. Here several studies were observational [25, [27], [28], [29], [30]], or the length of the interventions may not have been long enough to impact these variables. Interventions ranged from 3 to7 d [33, 38, 39, 41, 42, 44] to 24 wk [31], some of them being between 4 to 12 wk [32, [34], [35], [36], [37], 43]. Although human studies have shown alterations in gut microbiome diversity and composition that are sometimes correlated with host metabolic parameters, these effects were mostly small and inconsistent. As such, our current understanding of how the gut microbiome interacts with host energy metabolism is limited, and so is evidence of cause-and-effect relationships.

Additional limitations should be considered when establishing associations between gut microbiome and energy metabolism in humans, compared with animal models. Confounders that may affect both the gut microbiome and energy metabolism should be assessed with high quality tools or surrogate markers, such as dietary intake (e.g., collecting weighted food records), physical activity data (e.g., using accelerometers), and gut transit time (e.g., assessing stool consistency). The current evidence from animal models and the limited evidence from human studies can, however, be used to explore future hypotheses to fill these knowledge gaps.

## Future directions

**FIGURE 3:**
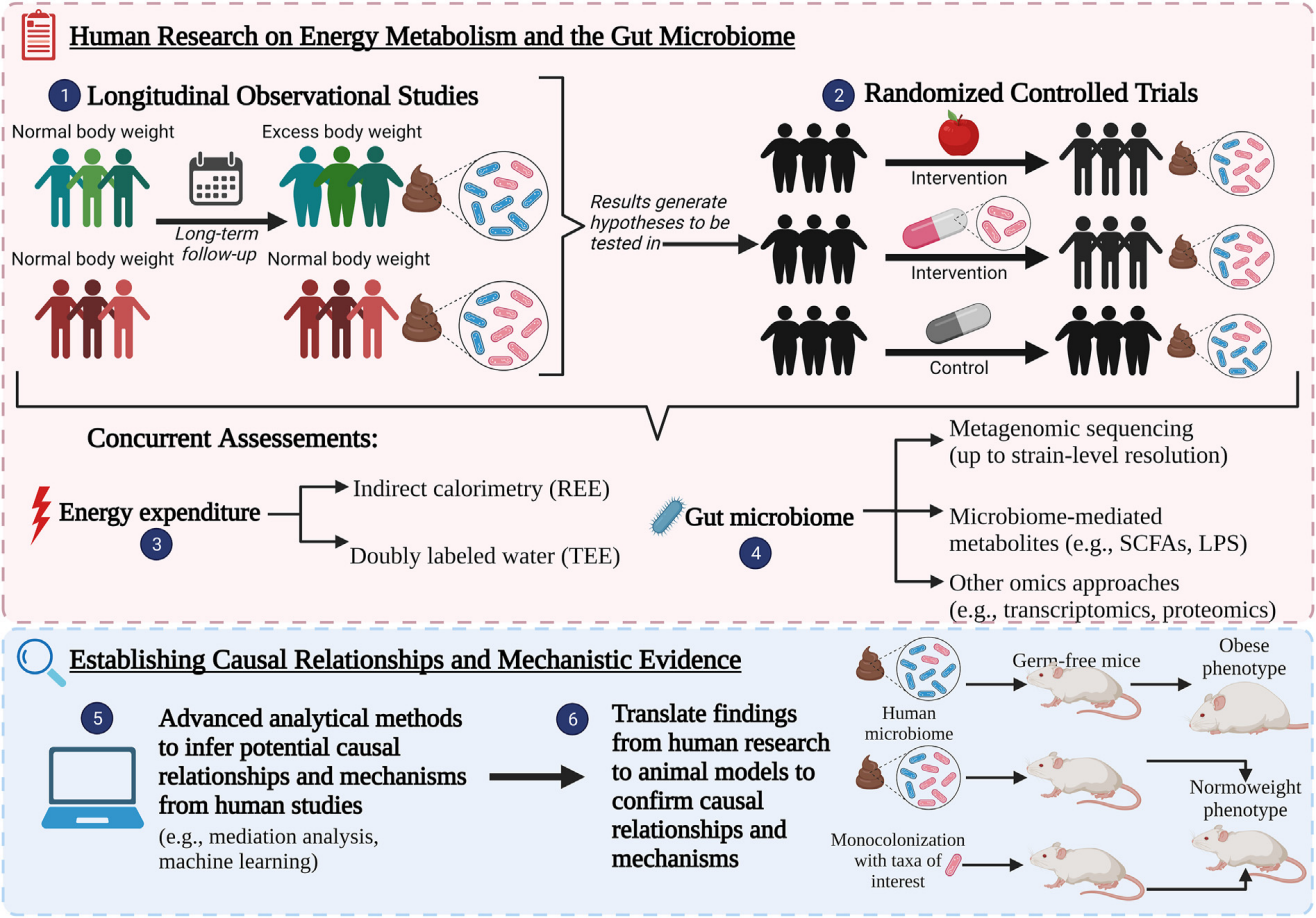
**Framework for future studies on the relation between energy metabolism and the gut microbiome.** 1) Longitudinal observational studies can be used to assess how gut microbiome changes in long term and how those changes are related to health outcomes; 2) Randomized controlled trials can test hypotheses generated from longitudinal studies in well-controlled settings; 3) Energy expenditure should be assessed by indirect calorimetry, preferably metabolic chamber, and/or doubly labeled water; 4) gut microbiome composition should be assessed by metagenomic sequencing and its functions should be assessed by analyzing its metabolites and by multiomics; 5) Results from human studies should be analyzed using advanced analytical methods to infer possible causations and mechanisms; 6) Those findings can be translated into animal models with human associated microbiome and monocolonization to confirm causation and mechanisms. Abbreviations: REE, resting energy expenditure; TEE, total energy expenditure; SCFAs, short-chain fatty acid; LPS, lipopolysaccharides.

An experimental framework for advancing research in gut microbiome and energy metabolism is summarized in Figure 3. Longitudinal observational cohort studies would be useful to understand how the microbiome and metabolic traits change in tandem and interact long term to produce different states of health and disease. These studies cannot determine causality or inform clinical practice. Nonetheless, multiomics approaches (e.g., metagenomics, metabolomics) can be used to deeply phenotype participant cohorts to inform future randomized controlled trials to test targeted nutritional strategies. Randomized controlled trials could specifically study how microbiome changes are linked to changes in REE and RER and if this leads to changes in body weight and body composition. In this scenario, a more rigorous approach must be used to prevent alterations in other factors that could influence metabolic outcomes, including modifications in diet, physical activity levels, and medications. Those confounders should be controlled for by using stratified randomization and/or adequately assessed to control for in the statistical analyses. Feeding trials are an important method to reduce dietary confounders and are useful to identify how specific foods or nutrients affect gut microbiome and energy metabolism; thus, informing possible causations that would need to be confirmed as suggested subsequently.

More robust methods to assess energy metabolism can also be used. Most of the reviewed studies used a metabolic cart for indirect calorimetry; however, metabolic chambers have higher precision [45]. Metabolic chambers also allow for analysis of different components of TEE, such as sleep, physical activity, and the thermic effect of food [45]. Energy expenditure can also be assessed in free living conditions using DLW [45] but only one study used this method [33].

It is not possible to assess direct mechanisms related to energy metabolism in humans. However, disease and mechanistic markers, as well as variables linked to the gut microbiome can be used to explore their effects on energy metabolism; for example, measuring SCFAs, LPS, and bile acids. Most studies have analyzed SCFA concentrations only in feces; combining the measurement of these compounds in the blood and in stool could provide additional insights into the number of SCFAs being produced and absorbed into the bloodstream. However, because SCFAs are also absorbed by colonocytes, it is not currently possible to know the absolute amount produced by the gut microbiome to assess energy harvest [9, 50]. Measuring the concentration of circulating LPS and inflammatory markers could also help elucidate the influence of gut microbiome on inflammation and, consequently, on energy metabolism. Analyzing bile acids in the feces and blood could be useful in identifying how the gut microbiome metabolize human bile acids and how they activate signaling pathways.

Causal inference in humans may be accomplished through advanced statistical approaches in well-controlled longitudinal observational or interventional studies. Mendelian randomization can be used to help establish causation by analyzing if natural genetic variants or microbial pathways influence the observed metabolic outcomes [79]. Mediation analysis can infer whether the microbiome mediates effects on the metabolic trait of interest [80]. The metadata acquired with metagenomic sequencing and multiomics approaches can also be used to identify associations between the microbiome and host phenotypes using machine learning models [81].

Another way to establish causation in humans is by performing FMT from healthy donors to recipients with specific health conditions to determine whether a ‘healthy’ gut microbiome leads to clinical improvements. An important limitation of this method is that healthy individuals cannot receive FMT from those with a disease/condition to determine if the microbiome of the latter contributes to the development of the disease/conditions.

Although using appropriate and robust study designs and methods can support our understanding of how the microbiome may mediate effects on energy metabolism, it is still not possible to determine causal relationships because of ethical and practical issues in humans. An alternative and potentially complementary strategy is using the human microbiome-associated mouse model. In this model, germ-free mice are inoculated with human microbiomes and are monitored for signs of disease development [82] or, in this case, alterations in energy metabolism. If species of interest are identified in human studies or in human microbiome-associated mouse models, monocolonization of germ-free mice (i.e., inoculating a single strain) can be used to elucidate the impact of such species on energy metabolism. However, the effect of a single microbe is artificial and would be different from the effects of community interactions [83].

In conclusion, the gut microbiome may be one modifiable factor that affects human energy metabolism. Observational studies have reported associations between microbial taxa and nutritional status that are related to energy metabolism, but findings were inconsistent. Some interventional studies aimed to modulate the gut microbiome to improve metabolic outcomes, including energy metabolism, through various approaches but were unable to define an ideal intervention. Therefore, the development of recommendations for modulating the gut microbiome to influence energy metabolism is still not possible. Rigorous research in humans that integrates multiomics approaches and advanced statistical analyses is required to inform the development of precision nutritional strategies that target health outcomes related to energy metabolism via gut microbiome modulation.

## Acknowledgments

Figures created with BioRender.com (https://biorender.com/). JFM was funded by the Alberta Diabetes Institute. CMP was funded by Campus Alberta Innovation Program in Nutrition, Food, and Health.

### Author contributions

JM, BPW, JW, CMP: were responsible for the design of the study; JM, AMA: performed the search and screening of papers included and data extraction; JM: wrote the manuscript; all authors reviewed and edited the manuscript; and all authors read and approved the final version.

### Author disclosures

ECD was previously employed by AgriFiber Solutions LLC, an agricultural technology company that manufactures upcycled dietary fibers and prebiotics. JW is further a co-owner of Synbiotics Health, a developer of synbiotic products. CMP reports receiving honoraria and/or paid consultancy from Abbott Nutrition, Nutricia, Nestle Health Science, Fresenius Kabi, Pfizer, and Helsinn. JW and CMP also received funding from Almased. Other authors report no conflict of interest.

## References

[1] Cani P.D., Delzenne N.M. (2009). The role of the gut microbiota in energy metabolism and metabolic disease, Curr. Pharm. Des.

[2] Alghannam A.F., Ghaith M.M., Alhussain M.H. (2021). Regulation of energy substrate metabolism in endurance exercise. Int. J. Environ. Res. Public. Health.

[3] Rigoulet M., Bouchez C.L., Paumard P., Ransac S., Cuvellier S., Duvezin-Caubet S. (2020). Cell energy metabolism: an update. Biochim. Biophys. Acta. Bioenerg.

[4] Galgani J., Ravussin E. (2008). Energy metabolism, fuel selection and body weight regulation. Int. J. Obes. (London).

[5] Soares M.J., Müller M.J. (2018). Resting energy expenditure and body composition: critical aspects for clinical nutrition, Eur. J. Clin. Nutr.

[6] Calcagno M., Kahleova H., Alwarith J., Burgess N.N., Flores R.A., Busta M.L. (2019). The thermic effect of food: a review. J. Am. Coll. Nutr.

[7] Jandhyala S.M., Talukar R., Subramanyan C., Vuyyuru H., Sasikata M., Reddy D.N. (2015). Role of the normal gut microbiota. World J. Gastroenterol.

[8] Morrison D.J., Preston T. (2016). Formation of short chain fatty acids by the gut microbiota and their impact on human metabolism. Gut. Microbes.

[9] Den Besten G., Eunen K.Van, Groen A.K., Venema K., Reijngoud D.–J., Bakker B.M. (2013). The role of short-chain fatty acids in the interplay between diet, gut microbiota, and host energy metabolism, J. Lipid. Res..

[10] Gao X., Lin S.H., Ren F., Li J.T., Chen J.J., Yao C.-B. (2016). Acetate functions as an epigenetic metabolite to promote lipid synthesis under hypoxia. Nat. Commun.

[11] Sahuri-Arisoylu M., Brody L.P., Parkinson J.R., Parkes H., Navaratnam N., Miller A.D. (2016). Reprogramming of hepatic fat accumulation and 'browning' of adipose tissue by the short-chain fatty acid acetate. Int. J. Obes. (London).

[12] Wang H., Ye J. (2015). Regulation of energy balance by inflammation: common theme in physiology and pathology. Rev. Endocr. Metab Disord.

[13] Furman D., Campisi J., Verdin E., Carrera-Bastos P., Targ S., Franceschi C. (2019). Chronic inflammation in the etiology of disease across the life span. Nat. Med.

[14] Bohan R., Tianyu X., Tiantian Z., Ruonan F., Hongtao H., Qiong W. (2019). Gut microbiota: a potential manipulator for host adipose tissue and energy metabolism. J. Nutr. Biochem.

[15] Boulangé C.L., Neves A.L., Chilloux J., Nicholson J.K., Dumas M.–E. (2016). Impact of the gut microbiota on inflammation, obesity, and metabolic disease. Genome Med.

[16] Jia W., Xie G., Jia W. (2018). Bile acid-microbiota crosstalk in gastrointestinal inflammation and carcinogenesis. Nat. Rev. Gastroenterol. Hepatol.

[17] Amabebe E., Robert F.O., Agbalalah T., Orubu E.S. (2020). Microbial dysbiosis-induced obesity: role of gut microbiota in homoeostasis of energy metabolism. Br. J. Nutr.

[18] Heiss C.N., Olofsson L.E. (2018). Gut microbiota-dependent modulation of energy metabolism J. Innate. Immun.

[19] Bakker G.J., Zhao J., Herrema H., Nieuwdorp M. (2015). Gut microbiota and energy expenditure in health and obesity. J. Clin. Gastroenterol.

[20] Rosenbaum M., Knight R., Leibel R.L. (2015). The gut microbiota in human energy homeostasis and obesity, Trends Endocrinol. Metabol.

[21] Vrieze A., Holleman F., Zoetendal E.G., De Vos W.M., Hoekstra J.B.L., Nieuwdorp M. (2010). The environment within: how gut microbiota may influence metabolism and body composition. Diabetologia.

[22] Singh R.K., Chang H.-W., Yan D., Lee K.M., Ucmak D., Wong K. (2017). Influence of diet on the gut microbiome and implications for human health. J. Transl. Med.

[23] World Health Organization (2010). https://www.who.int/europe/news-room/fact-sheets/item/a-healthy-lifestyle-who-recommendations.

[24] World Health Organization (2021). https://www.who.int/news-room/fact-sheets/detail/obesity-and-overweight.

[25] Bielik V., Hric I., Baláž V., Penesová A., Vávrová S., Grones J. (2020). Gut microbiota diversity in lean athletes is associated with positive energy balance. Ann. Nutr. Metab.

[26] Boekhorst J., Venlet N., Procházková N., Hansen M.L., Lieberoth C.B., Bahl M.I. (2022). Stool energy density is positively correlated to intestinal transit time and related to microbial enterotypes. Microbiome.

[27] Ghosh T.S., Gupta S.S., Bhattacharya T., Yadav D., Barik A., Chowdhury A. (2014). Gut microbiomes of indian children of varying nutritional status. PLoS ONE.

[28] Goffredo M., Mass K., Parks E.J., Wagner D.A., Mcclure E.A., Graf J. (2016). Role of gut microbiota and short chain fatty acids in modulating energy harvest and fat partitioning in youth. J. Clin. Endocrinol. Metab.

[29] Wan Y., Yuan J., Li J., Li H., Yin K., Wang F. (2020). Overweight and underweight status are linked to specific gut microbiota and intestinal tricarboxylic acid cycle intermediates. Clin. Nutr.

[30] Yun Y., Kim H.-N., Kim S.E., Heo S.G., Chang Y., Ryu S. (2017). Comparative analysis of gut microbiota associated with body mass index in a large Korean cohort. BMC Microbiol..

[31] Bendtsen L.Q., Blædel T., Holm J.B., Lorenzen J.K., Mark A.B., Kiilerich P. (2018). High intake of dairy during energy restriction does not affect energy balance or the intestinal microflora compared with low dairy intake in overweight individuals in a randomized controlled trial, Appl. Physiol. Nutr. Metab.

[32] Canfora E.E., Van Der Beek C.M., Hermes G.D.A., Goossens G.H., Jocken J.W.E., Holst J.J. (2017). Supplementation of diet with galacto-oligosaccharides increases bifidobacteria, but not insulin sensitivity, in obese prediabetic individuals. Gastroenterology.

[33] Karl J.P., Margolis L.M., Madslien E.H., Murphy N.E., Castellani J.W., Gundersen Y. (2017). Changes in intestinal microbiota composition and metabolism coincide with increased intestinal permeability in young adults under prolonged physiological stress. Am. J. Physiol. Gastrointest. Liver Physiol.

[34] Karl J.P., Meydani M., Barnett J.B., Vanegas S.M., Goldin B., Kane A. (2017). Substituting whole grains for refined grains in a 6-wk randomized trial favorably affects energy-balance metrics in healthy men and postmenopausal women, Am. J. Clin. Nutr.

[35] Most J., Penders J., Lucchesi M., Goossens G.H., Blaak E.E. (2017). Gut microbiota composition in relation to the metabolic response to 12-week combined polyphenol supplementation in overweight men and women, Eur. J. Clin. Nutr.

[36] Müller M., Hermes G.D.A., Emanuel E.C., Holst J.J., Zoetendal E.G., Smidt H. (2020). Effect of wheat bran derived prebiotic supplementation on gastrointestinal transit, gut microbiota, and metabolic health: a randomized controlled trial in healthy adults with a slow gut transit. Gut. Microbes.

[37] Yu E.W., Gao L., Stastka P., Cheney M.C., Mahabamunuge J., Torres Soto M. (2020). Fecal microbiota transplantation for the improvement of metabolism in obesity: the FMT-TRIM double-blind placebo-controlled pilot trial. PLOS Medicine.

[38] Basolo A., Hohenadel M., Ang Q.Y., Piaggi P., Heinitz S., Walter M. (2020). Effects of underfeeding and oral vancomycin on gut microbiome and nutrient absorption in humans. Nat. Med.

[39] Jumpertz R., Le D.S., Turnbaugh P.J., Trinidad C., Bogardus C., Gordon J.I. (2011). Energy-balance studies reveal associations between gut microbes, caloric load, and nutrient absorption in humans, Am. J. Clin. Nutr.

[40] Kaczmarek J.L., Liu X., Charron C.S., Novotny J.A., Jeffery E.H., Seifried H.E. (2019). Broccoli consumption affects the human gastrointestinal microbiota. J. Nutr. Biochem.

[41] Karusheva Y., Koessler T., Strassburger K., Markgraf D., Mastrototaro L., Jelenik T. (2019). Short-term dietary reduction of branched-chain amino acids reduces meal-induced insulin secretion and modifies microbiome composition in type 2 diabetes: a randomized controlled crossover trial, Am. J. Clin. Nutr.

[42] Papadopoulou R.T., Theodorou M.R., Ieong C.S., Ballantyne K., Marshall D., Verney A. (2020). The acute effect of meal timing on the gut microbiome and the cardiometabolic health of the host: a crossover randomized control trial. Ann. Nutr. Metabol.

[43] Kelder T., Stroeve J.H.M., Bijlsma S., Radonjic M., Roeselers G. (2014). Correlation network analysis reveals relationships between diet-induced changes in human gut microbiota and metabolic health. Nutr. Diabetes.

[44] Ott B., Skurk T., Lagkouvardos L., Fischer S., Büttner J., Lichtenegger M. (2018). Short-term overfeeding with dairy cream does not modify gut permeability, the fecal microbiota, or glucose metabolism in young healthy men. J. Nutr.

[45] Mtaweh H., Tuira L., Floh A.A., Parshuram C.S. (2018). Indirect calorimetry: history, technology, and application. Front. Pediatr.

[46] Sanchez-Delgado G., Ravussin E. (2020). Assessment of energy expenditure: are calories measured differently for different diets?. Curr. Opin. Clin. Nutr. Metab. Care.

[47] Westerterp K.R. (2017). Doubly labelled water assessment of energy expenditure: principle, practice, and promise. Eur. J. Appl. Physiol.

[48] Basolo A., Parrington S., Ando T., Hollstein T., Piaggi P., Krakoff J. (2020). Procedures for measuring excreted and ingested calories to assess nutrient absorption using bomb calorimetry. Obesity.

[49] Holscher H.D. (2017). Dietary fiber and prebiotics and the gastrointestinal microbiota. Gut Microbes.

[50] Primec M., Mičetić-Turk D., Langerholc T. (2017). Analysis of short-chain fatty acids in human feces: a scoping review. Anal. Biochem.

[51] Jacobsen R., Lorenzen J.K., Toubro S., Krog-Mikkelsen I., Astrup A. (2005). Effect of short-term high dietary calcium intake on 24-h energy expenditure, fat oxidation, and fecal fat excretion. Int. J. Obes.

[52] Nakamura T., Takebe K., Tando Y., Arai Y., Yamada N., Ishii M. (1995). Faecal triglycerides and fatty acids in the differential diagnosis of pancreatic insufficiency and intestinal malabsorption in patients with low fat intakes. J. Int. Med. Res..

[53] Tyler A.D., Smith M.I., Silverberg M.S. (2014). Analyzing the human microbiome: a “how to” guide for physicians. Am. J. Gastroenterol.

[54] Prakash T., Taylor T.D. (2012). Functional assignment of metagenomic data: challenges and applications. Brief. Bioinform.

[55] Heintz-Buschart A., Wilmes P. (2018). Human gut microbiome: function matters. Trends Microbiol..

[56] Kim K.N., Yao Y., Ju S.Y. (2019). Short chain fatty acids and fecal microbiota abundance in humans with obesity: a systematic review and meta-analysis. Nutrients.

[57] Shim J.–S., Oh K., Kim H.C. (2014). Dietary assessment methods in epidemiologic studies. Epidemiol Health.

[58] Mueller N.T., Bakacs E., Combellick J., Grigoryan Z., Dominguez-Bello M.G. (2015). The infant microbiome development: mom matters. Trends Mol. Med.

[59] Ma L., Terwilliger A., Maresso A.W. (2015). Iron and zinc exploitation during bacterial pathogenesis. Metallomics.

[60] Kim S., Covington A., Pamer E.G. (2017). The intestinal microbiota: Antibiotics, colonization resistance, and enteric pathogens. Immunol. Rev..

[61] Burt D.G., Lamb K., Nicholas C., Twist C. (2014). Effects of exercise-induced muscle damage on resting metabolic rate, sub-maximal running and post-exercise oxygen consumption, Eur. J. Sport Sci..

[62] Shanahan E.R., Mcmaster J.J., Staudacher H.M. (2021). Conducting research on diet–microbiome interactions: a review of current challenges, essential methodological principles, and recommendations for best practice in study design. J. Hum. Nutr. Diet.

[63] Wahlström A., Sayin S.I., Marschall H.–U., Bäckhed F. (2016). Intestinal crosstalk between bile acids and microbiota and its impact on host metabolism. Cell Metab.

[64] Wu Y., Shen Y., Zhu Y., Mupunga J., Zou L., Liu C. (2019). Broccoli ingestion increases the glucosinolate hydrolysis activity of microbiota in the mouse gut. Int. J. Food Sci. Nutr.

[65] Bouranis J.A., Beaver L.M., Ho E. (2021). Metabolic fate of dietary glucosinolates and their metabolites: a role for the microbiome. Front. Nutr.

[66] Palafox-Carlos H., Ayala-Zavala J.F., González-Aguilar G.A. (2011). The role of dietary fiber in the bioaccessibility and bioavailability of fruit and vegetable antioxidants. J. Food Sci..

[67] Huttenhower C., Gevers D., Knight R., Abubucker S., Badger J.H., Chinwalla A.T. (2012). Structure, function and diversity of the healthy human microbiome. Nature.

[68] Valdés L., Cuervo A., Salazar N., Ruas-Madiedo P., Gueimonde M., González S. (2015). The relationship between phenolic compounds from diet and microbiota: impact on human health. Food Funct.

[69] Villegas-Aguilar M.D.C., Fernández-Ochoa Á., Cádiz-Gurrea M.D.L.L., Pimentel-Moral S., Lozano-Sánchez J., Arráez-Román D. (2020). Pleiotropic biological effects of dietary phenolic compounds and their metabolites on energy metabolism, inflammation and aging. Molecules.

[70] Bifari F., Ruocco C., Decimo I., Fumagalli G., Valerio A., Nisoli E. (2017). Amino acid supplements and metabolic health: a potential interplay between intestinal microbiota and systems control. Genes Nutrit.

[71] Wu G.D., Chen J., Hoffmann C., Bittinger K., Chen Y.–Y., Keilbaugh S.A. (2011). Linking long-term dietary patterns with gut microbial enterotypes. Science.

[72] Zou H., Wang D., Ren H., Cai K., Chen P., Fang C. (2020). Effect of caloric restriction on bmi, gut microbiota, and blood amino acid levels in non-obese adults. Nutrients.

[73] Gorvitovskaia A., Holmes S.P., Huse S.M. (2016). Interpreting Prevotella and Bacteroides as biomarkers of diet and lifestyle. Microbiome.

[74] Hjorth M.F., Blædel T., Bendtsen L.Q., Lorenzen J.K., Holm J.B., Kiilerich P. (2019). Prevotella-to-Bacteroides ratio predicts body weight and fat loss success on 24-week diets varying in macronutrient composition and dietary fiber: results from a post-hoc analysis. Int. J. Obes.

[75] Hjorth M.F., Roager H.M., Larsen T.M., Poulsen S.K., Licht T.R., Bahl M.I. (2018). Pre-treatment microbial Prevotella-to-Bacteroides ratio, determines body fat loss success during a 6-month randomized controlled diet intervention. Int. J. Obes.

[76] Turnbaugh P.J., Ley R.E., Mahowald M.A., Magrini V., Mardis E.R., Gordon J.I. (2006). An obesity-associated gut microbiome with increased capacity for energy harvest. Nature.

[77] Sze M.A., Schloss P.D., Fraser C.M. (2016). Looking for a signal in the noise: revisiting obesity and the microbiome. mBio.

[78] Magne F., Gotteland M., Gauthier L., Zazueta A., Pesoa S., Navarrete P. (2020). The firmicutes/bacteroidetes ratio: a relevant marker of gut dysbiosis in obese patients?. Nutrients.

[79] Sanna S., Van Zuydam N.R., Mahajan A., Kurilshikov A., Vich Vila A., Võsa U. (2019). Causal relationships among the gut microbiome, short-chain fatty acids and metabolic diseases. Nat. Genet..

[80] Zhang H., Chen J., Li Z., Liu L. (2021). Testing for mediation effect with application to human microbiome data. Stat. Biosci.

[81] Namkung J. (2020). Machine learning methods for microbiome studies. J. Microbiol..

[82] Walter J., Armet A.M., Finlay B.B., Shanahan F. (2020). Establishing or exaggerating causality for the gut microbiome: lessons from human microbiota-associated rodents. Cell..

[83] Round J.L., Palm N.W. (2018). Causal effects of the microbiota on immune-mediated diseases. Sci. Immunol.

